# Recruitment and retention of adolescents for an ecological momentary assessment measurement burst mental health study: The MHIM engagement strategy

**DOI:** 10.1111/hex.14065

**Published:** 2024-05-06

**Authors:** Aja L. Murray, Tong Xie, Luke Power, Lucy Condon

**Affiliations:** ^1^ Department of Psychology University of Edinburgh Edinburgh UK; ^2^ Faculty of Psychology Beijing Normal University Beijing China; ^3^ School of Social and Political Science University of Edinburgh Edinburgh UK; ^4^ National Institute for Health and Care Research Applied Research Collaboration West Bristol UK

**Keywords:** adolescent, attrition, ecological momentary assessment, longitudinal, measurement burst, retention

## Abstract

**Introduction:**

Recruitment and long‐term retention of adolescent participants in longitudinal research are challenging and may be especially so in studies involving remote measurement and biosampling components. The ability to effectively recruit and retain participants can be supported by the use of specific evidence‐based engagement strategies that are built in from the earliest stages.

**Methods:**

Informed by a review of the evidence on effective engagement strategies and consultations with adolescents (via two Young Person Advisory Groups [YPAGs]; ages 11–13 and 14–17), the current protocol describes the planned participant engagement strategy for the Mental Health in the Moment Study: a multimodal measurement burst study of adolescent mental health across ages 11–19.

**Results:**

The protocol incorporates engagement strategies in four key domains: consultations/co‐design with the target population, incentives, relationship‐building and burden/barrier reduction. In addition to describing general engagement strategies in longitudinal studies, we also discuss specific concerns regarding engagement in data collection methods such as biosampling and ecological momentary assessment where a paucity of evidence exists.

**Conclusion:**

Engagement strategies for adolescent mental health studies should be based on existing evidence and consultations with adolescents. We present our approach in developing the planned engagement strategies and also discuss limitations and future directions in engaging adolescents in longitudinal research.

**Patient or Public Contribution:**

The study design for this project places a strong emphasis on the active engagement of adolescents throughout its development. Specifically, the feedback and suggestions provided by the YPAGs have been instrumental in refining our strategies for maximising the recruitment and retention of participants.

## 1 INTRODUCTION

Gathering longitudinal data on mental health and its influences in adolescence is invaluable for illuminating its development and influences during and beyond this sensitive period.^1^, ^2^, ^3^ This information can guide strategies as to how and when to intervene to support lifelong mental health. Embedding real‐time methods such as ecological momentary assessment (EMA) that repeatedly sample individuals' behaviours and experiences within their natural settings, alongside passive data collection that captures data without active participation from individuals,^4^ allows one to capture symptoms and their variations and influences as they occur in an individual's ecological context. This subsequently permits the development of ‘ecologically embedded’ interventions.^5^ In the longer term, gathering this type of data over repeated bursts can provide insights into how day‐to‐day experiences impact long‐term mental health development and vice versa. When longitudinal surveys are combined with biosampling, this further allows insights into questions such as the biological impacts of experiences and biological influences on mental health over development.^6^ These considerations have been central to the design of the Mental Health in the Moment (MHIM) study: a longitudinal EMA measurement burst study of adolescent mental health.^7^ However, a key challenge is successfully recruiting and retaining adolescent participants to engage with intensive data collection over extended periods of time. The current protocol describes MHIM's participant engagement strategy, including plans for baseline recruitment and retention over 10 measurement bursts in a 5‐year data collection period.

**Figure 1:**
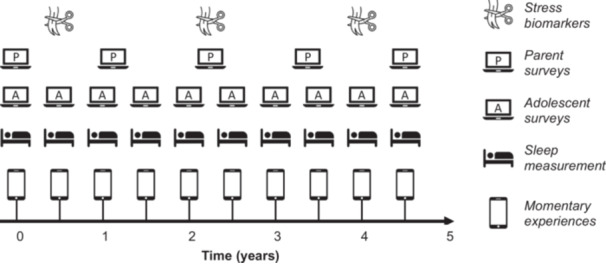
The data collection schedule for the Mental Health in the Moment Study.

MHIM is a planned measurement burst EMA study with online surveys and EMA bursts completed every 6 months by adolescent participants, parent online surveys completed on an annual basis and hair samples collected at three key measurement points over a 5‐year data collection period (see Figure 1). The online self‐report surveys and parental online surveys will measure mental health outcomes and influences that vary over timescales of months to years, whereas the EMA components measure mental health‐related concepts varying over hours to days. In the EMA component, young people will complete short sets of questions on their smartphone multiple times a day. These questions will be about their most recent experiences that day, for example, their affective state, and will occur over a 2‐week period. If young people deem this acceptable, we will also collect GPS co‐ordinates during the EMA data collection to examine the links between outcomes such as affective state and concurrent physical environmental factors such as weather and green/blue space. The EMA data collection will be coupled with daily sleep measurement, facilitated via radar‐based technology built into bedside devices. These devices were selected over traditional wrist‐worn actigraphs to minimise the day‐to‐day burden of wearing a device and enable the derivation of indices of sleep duration and quality. The hair samples will be analysed to establish biomarkers of cumulative stress (e.g., hair cortisol and cortisone) over periods that correspond with the EMA data collection. MHIM uses an accelerated cohort design,^8^ recruiting five age‐based cohorts with ages of 11, 12, 13, 14 and 15 at baseline who will be followed over 5 years. This means that the five age cohorts will start at the same time, which will allow ‘developmental analyses’ of the age range 11–19. However, it is important to note that this is dependent upon an assumption of cohort invariance. Otherwise, each age cohort can be analysed on its own as a traditional longitudinal cohort study.

Recruitment and retention in longitudinal studies of adolescence, such as the MHIM study, present multiple challenges. These include the competing concerns and busy schedules of adolescents, keeping in touch following school moves and after leaving school, obtaining parental as well as adolescent buy‐in and creating engagement strategies that are responsive to developmental changes occurring over the period of adolescence.^9^ Poor recruitment and retention can result in lower sample sizes, increased study costs and bias inferences when participation is selective with respect to the characteristics under study.^10^, ^11^

These difficulties may be further compounded in designs involving remote measurement such as EMA. For example, issues associated with remote measurement studies include the use of smartphones for intensive data collection, wearing or carrying additional devices, privacy concerns around location monitoring, inequalities in access to technologies when participants are expected to use their own devices and technological issues such as noncompatibility of data collection applications with participants' devices, battery drainage or other bugs. These are problematic as they can constitute additional burdens and barriers to participation.^12^, ^13^, ^14^, ^15^, ^16^, ^17^, ^18^ Participants may also have difficulty adhering to the intensive prompt schedule, for example, due to forgetting, incompatibility with their schedule or excessive burden.^19^

Established engagement strategies may not adapt well to EMA. For example, relationship‐building has been cited as an important factor in recruitment and retention of adolescents^20^; however, this may be more difficult when data collection occurs remotely. Young people may also be prone to ‘digital overload’, meaning that reminders and notifications can be counterproductive if used in excess.^21^, ^22^ Some recent studies have illustrated the hesitancy of participants to take part in EMA compared to other research designs.^23^, ^24^ For example, one study found that among a longitudinal cohort of late adolescents, only around 50% were willing to take part in an EMA study, despite generous incentives, and this was correlated with respondent characteristics.^23^ This suggests that despite the high compliance rates often achieved in EMA studies (∼75%–82% of prompts completed),^25^, ^26^ samples may be selective.

Biosampling studies may also face increased engagement challenges due to participant discomfort with providing samples, highlighting the importance of convenient collection methods that minimise participant burden and promote compliance.^27^ Measuring stress from hair samples requires either researchers or participants themselves (with help) to collect 100–150 strands of hair near the scalp.^28^, ^29^ Hair samples offer advantages over blood or saliva samples as they are relatively noninvasive, do not require medical professionals or a clinical environment and can be easily stored.^30^, ^31^ A survey of people living with HIV found that respondents were 1.5 times more likely to donate hair rather than blood, urine, saliva or stool for research.^32^ Additionally, hair samples provide scientific advantages in capturing concepts such as stress because they measure chronic physiological stress accumulated over periods of weeks and months, as compared to saliva‐, blood‐ or urine‐based measures, which provide momentary markers that are vulnerable to the effects of time‐of‐day and other within‐day fluctuations.^33^ Nevertheless, it is imperative for researchers to understand the challenges associated with the acceptability and feasibility of hair sample collection.^34^, ^35^ As hair samples need ideally to be collected near the scalp, participants may decline to have their hair cut due to specific hair stylings such as braids or dreadlocks or due to general concerns about how this may impact their appearance.^34^, ^35^ Hair collection also involves unique challenges for participants across different ethnicities and cultures, as hair may be associated with religious or spiritual beliefs.^36^, ^37^ Concerns about data privacy were also reported as a reason for refusal.^35^

Given the complexities of sustaining engagement in longitudinal studies with remote measurement and biosampling components, a robust strategy embedded from the earliest stages of the study is required to ensure the successful recruitment and retention of adolescent participants in these designs. Further, transparent reporting of the strategy can help maximise the insights gained for the field in terms of effective engagement strategies.^38^

The MHIM recruitment and retention strategy (summarised in Table 1) is informed by a narrative review of the literature focused on promising strategies for engaging adolescent participants in longitudinal health research, particularly for studies using remote measurement technologies such as EMA.^9^ The review showed that there is little direct evidence for what works; however, it highlighted four broad types of strategies considered promising: incentivising participation, reducing barriers and burdens, building positive relationships with participants and consultations with representatives of the target participant group. Our strategy is additionally informed by consultations with young people, whom we asked for input on the study design, including a consultation dedicated specifically to our recruitment and retention strategy.

**Table 1:** Summary of key engagement strategies components.

Domain	Strategies
Co‐production	− Consult YPAG on the factors to be included in the study to ensure it includes research questions relevant for them − Consult YPAG on engagement strategy components including incentives, reducing barriers and burdens, and building positive relationships at an early stage of study design − Consult YPAG on the specifics of the engagement strategy components within the context of a draft study protocol and materials (e.g., incentives and individual‐level feedback offered, measurement schedule, data collection application and measurement technologies), as well as on other aspects of the study, throughout the project's lifecycle − Make it clear to prospective participants that the study has been designed in consultation with people their age in the recruitment materials
Incentives	− Offer incentives to adolescent participants to take part − Offer sessions to schools/classes on mental health designed to help people learn more about the topic of mental health − Offer school‐level feedback on the data gathered − Offer guidance/sessions on how participants can reflect their research participation experiences in personal statements and curriculum vitae − Embed ‘mental health facts’ (selected to minimise the impact on responses) in the data collection application − Scale incentives to the amount of participation, including the response rate achieved in the EMA measurement outside of school hours, and therefore provide the incentives after (EMA) survey completion − Provide some individual‐level feedback, such as visualisations of responses, response rates or sleep patterns, selected to minimise the impact on responses − Considering offering the opportunity to keep measurement devices for participants who stay with the study until the end of the 5‐year data collection
Barrier/burden reduction	− Minimise technological barriers by using tried and tested and easy‐to‐use software/hardware, ensuring wide device compatibility (participants use their own devices where possible but study smartphones provided otherwise), minimal burden (e.g., charging/carrying of devices) and careful piloting to identify and address sources of burden − Very clearly explain issues such as confidentiality and anonymity to participants in the recruitment materials, assuming minimal prior knowledge and ensuring concerns are answered − Parental participation will not be an inclusion criterion for adolescent participation − Data collection will aim to avoid exam times − Participants will be sent reminders to complete the surveys − Participants will use their own smartphones (smartphones will be provided where participants do not have access to a phone) − A window of up to 2 h will be given to allow responses to prompts (the time of responding rather than prompt sent will be used in analyses) − The target number of EMA prompts/day will be communicated as 3–4, though participants may be issued more − Participants will be informed that they can miss prompts if they need to − The researchers will seek to negotiate a time within the school day for participants to complete the initial on‐boarding and online survey − The in‐person data collection components will take place at participants' schools to minimise any travel burden and the majority of the study will otherwise be completed remotely − Participants will not be asked to commit to all 5 years of the study in one go (participation will be renewed annually)
Relationships	− Select study staff based partly on an assessment of ability to build rapport with adolescents − Provide training to study staff in building rapport with adolescents − Visit schools to present the project to prospective participants − Ensure that the value and importance of participants to the study is clearly conveyed in communications with prospective participants − Study staff will collect the hair samples to provide an in‐person contact opportunity, rather than deliver this component via self‐collection
Other	− Use statistical approaches to mitigate bias and precision loss due to selective participation and attrition − Gather data and conduct analyses that can advance knowledge of effective engagement strategies for the field

Abbreviations: EMA, ecological momentary assessment; YPAG, Young Person Advisory Group.

## 2 THE MHIM STRATEGY

### 2.1 Co‐design with adolescents

There is increasing recognition of an ethical responsibility to consult young people on matters that affect them, including on the design of research studies about them.^39^, ^40^, ^41^ Previous research in both longitudinal studies of adolescence^16^, ^42^ and remote measurement designs^17^, ^43^ have further highlighted the value of consulting with representatives of the target population in their study design and recruitment and retention strategy development.

Based on these rationales, the MHIM study design and engagement strategy is informed by consultations with young people as advisors. Two groups of 5 young people (aged 11–13 and 14–17) were consulted and provided invaluable input on the engagement strategy. These young people were part of Bristol's Generation R Young Person's Advisory Group (YPAG). The Bristol YPAG recruited young professional (YP) through an open application process complemented by diverse promotional channels (e.g., research talks in schools, charity activities) to ensure a broad representation of youth. Before joining the YPAG, both YP and their parents were provided with consent/assent forms and received comprehensive induction sessions addressing any queries they had. The panel of YP for this specific project was recruited from the Bristol YPAG by emailing the group with an introduction of the project and the tasks involved. Selection criteria were solely based on the age requirements. We focused on these age groups because the MHIM sample will be aged 11–15 at baseline. Further consultations with older YP are planned to inform strategies for keeping MHIM participants engaged in later years of the study. The consultation with each group took place online and lasted 1 h. During the session, the researchers introduced the project, provided opportunities to ask questions and asked a set of discussion questions regarding engagement strategies in five themes (including ‘other’). As YP acted as advisors sharing knowledge and opinions drawn from their experiences rather than assuming the role of research participants, ethical approval was not pursued in alignment with relevant guidelines.^40^, ^44^ As the session was part of a series of YPAG with the same young people, the session also included brief feedback on how their earlier input had influenced our mental health research. They were offered a £30 shopping voucher as compensation for their time. Their input is reflected in the sections that follow and in the engagement strategy outlined in Table 1. These recommendations primarily reflect areas of consensus within and across the YPAGs, and there was generally a high degree of consistency in the views expressed by the young people of different ages; however, we have also reflected in places where there were differences of opinion. No formal decision‐making process was implemented (e.g., vote counting). Rather, all views expressed were recorded, and where there was consensus or where a view was expressed and not opposed, this was adopted into our strategy in most cases. In a small number of cases, suggestions were not adopted (e.g., a desire for individual‐level feedback on stress and mental health) based on scientific or practical considerations. In other cases where there was disagreement among the young people, both views were considered, and a decision was made based on balancing the different considerations raised by young people. The views expressed by young people and how they influenced the study (or why they were not adopted, in a small number of cases) are summarised in Supporting Information S1: Table 1.

The YPAG generally endorsed the importance of consulting with young people in designing a project, with members suggesting that they would be more likely to take part in a research study as a participant if they knew that people their age had been involved in its design. The same YPAGs provided earlier input on the research questions of the study reflecting the value and importance of engaging young people from the earliest stages of a project's lifecycle.

### 2.2 Incentives

Providing incentives has been consistently shown to be one of the most effective strategies for increasing response rates and retention across different research designs and populations.^45^, ^46^ There is also some evidence that informs optimal schedules of incentives. For example, unconditional monetary incentives provided before participation have been shown to achieve the same or higher retention than incentives paid after participation,^47^, ^48^, ^49^ suggesting that costs can be reduced by offering advance payment of incentives. In principle this could also help with the underrepresentation of less privileged groups who may be discouraged from participating where there is a need to wait for payment. In terms of the level and type of incentive, research indicates that monetary incentives are generally more effective than other types of incentives, including vouchers.^45^ Higher values of incentives appear to increase participation^50^; however, there is an issue with ‘diminishing returns’ and the larger ethical concern regarding incentives becoming coercive. Further, in some contexts it is important to consider the impact of incentives on benefits (social security) eligibility for participants. Recent research indicates that adolescents feel that £10/h is a fair reward for taking part in longitudinal research.^16^ Finally, evidence suggests that additional incentives (e.g., increased monetary incentives) can be used as a strategy to increase the participation of those who may be more difficult to secure.^51^, ^52^

A small number of studies have also examined incentive strategies specifically within EMA designs. A recent meta‐analysis found that there were better average compliance rates in studies using monetary incentives (82.2%) than nonmonetary incentives (77.5%) or no incentives (76.2%); however, beyond this, there was little evidence for any specific strategies (e.g., offering bonus incentives) having a substantial effect.^26^ There is, however, some within‐study evidence that for the same value of incentive, receiving the reward for each completed survey rather than as an accumulated amount at the end of a week is more effective.^53^ It is also important to consider that some strategies could have a paradoxical counterproductive effect. One recent study also highlighted the possibility that some strategies can have a negative effect, for example, in a student alcohol use study, compliance declined immediately following the award of bonuses.^54^ There is little evidence regarding the effects of leveraging incentives in biosampling research. In one qualitative interview study exploring the perspectives of underrepresented participants concerning their involvement in biospecimen research, approximately half of the participants reported monetary incentives as a motivating factor for providing hair samples.^55^

Our YPAG had mixed views about incentives, with some of the younger members suggesting that it may be better not to provide monetary incentives to participants as, while it may motivate participation, they felt it would be better to recruit intrinsically motivated participants to obtain higher quality data. The older YPAG generally felt that incentives would be appropriate and that these should be scaled to response rates. In essence, young people should receive a larger incentive for taking part in more components of the research and responding to more of the EMA prompts outside of school hours. They also highlighted some alternative ways to incentivise participation, such as providing coaching on how to reflect research participation in personal statements and providing feedback on responses. There was some support for the idea that providing feedback to schools may also impact individual‐level participation motivation; however, this was not considered to be as interesting for prospective participants as individual‐level feedback. A related motivating factor mentioned was the possibility of learning more about mental health. We identified several possible ways to achieve this, including embedding information about mental health into the data collection flow and providing workshops in schools. Finally, it was noted that getting to keep the sleep measurement devices would be a motivating factor. The incentive‐based components of MHIM in view of the evidence and YPAG views are outlined in Table 1. These include offering sessions on the topic of mental health, school‐level feedback based on the data collected, guidance on how participants can reflect their participation in CVs and personal statements for university/job applications, embedding information about mental health in an engaging way within the study flow, scaling incentives to the amount of data provided, providing some individual‐level feedback designed to minimise the impact of feedback on behaviour/responses and considering providing the opportunity to keep the sleep measurement devices if participants manage to stay with the study for its entire period of data collection.

### 2.3 Relationship‐building

Though more difficult to measure and quantify in terms of its effects on engagement, there is consensus among longitudinal researchers regarding the importance of building positive relationships with participants to motivate their engagement.^9^ For studies that are largely conducted remotely, however, building relationships with participants can be more challenging due to a lack of face‐to‐face contact. One possible solution is to attempt to recreate these relationship‐building activities online. For example, one EMA study of a sample of men who have sex with men found that participants recruited online versus in‐person did not differ in their response rates^56^ when they completed an on‐boarding session with study staff via videoconferencing rather than in‐person.

Our YPAG pointed to the importance of relationships in various ways, noting that they would like researchers to come to their school to talk about the study. They also indicated that how much they liked the researchers would influence their participation. However, it was mentioned that keeping in touch with the researchers after this point would be less important. In fact, some YPAG members raised the interesting issue that if they are providing sensitive information, it may be counterproductive to get to know the researchers too much as this might undermine their comfort with providing sensitive information. Otherwise, the YPAG mentioned some ways in which researchers might convey how much participants are valued in a study (as well as making this explicit), such as by offering incentives, providing certificates and explaining the impact of participants' contributions to answering important scientific questions. The younger YPAG also noted the importance of knowing background information about the researchers to assess the legitimacy of a study (though they would trust an invitation to a research study that came via their school or parents). MHIM considers positive relationship‐building in several ways in its recruitment and retention strategy, as outlined in Table 1. These include considering the ability to build rapport with adolescents as part of the selection criterion for study staff, providing training to staff in building rapport with adolescents and visiting schools to present the project to prospective participants. This latter strategy, however, depends on the capacity and preference of the schools as it can be administratively burdensome to receive permission for visitors in schools. In some contexts, visitors in schools may not be possible at all.

### 2.4 Barrier minimisation

There are a wide range of potential barriers to research participation. These could include mobility constraints, a lack of trust or interest or perceived benefit of the research, or limited time.^9^ One of the most critical considerations, however, is ensuring that on‐boarding and data collection is not overly burdensome. Given the intensity of the data collection method, EMA data collection parameters are particularly key. Previous EMA studies have found that willingness to take part is greatest when data collection is less burdensome^57^ and participants' perception of data collection ease is correlated with their compliance.^58^

Some previous studies have explored the effects of patterns, frequency and lengths of data collections in EMA surveys; however, findings have been mixed.^26^, ^59^, ^60^, ^61^, ^62^, ^63^ A recent comprehensive systematic review of EMA studies found no relation between EMA survey length, frequency or study duration and compliance.^26^ This is consistent with experimental studies^64^, ^65^ or survey length,^65^ though the latter study found that increasing the survey length did reduce within‐person variability and relations of variables. Overall, it is difficult to draw firm conclusions on the optimal number of prompts per day and survey length to promote compliance and achieve minimum numbers of observations for statistical analyses.

Other studies have found that EMA compliance is related to how easy participants find it to take part, the usefulness of the compliance portal where they could view their response rates and the timely resolution of issues,^58^ pointing to the importance of attending to user experience considerations and providing adequate troubleshooting support for compliance in EMA studies. A systematic review of EMA studies in depressive patients also found that compliance was higher when participants used their own smartphones.^13^ The latter finding is consistent with evidence that EMA participants prefer not to carry and have to keep multiple devices charged.^12^ Finally, focus group findings suggest that an easy‐to‐use application that does not overload respondents with notifications is perceived by young people to be important.^16^

Regarding the hair sampling component, feasibility studies conducted among children and adolescents have shown that hair sample collection was largely acceptable with the consent rates ranging from 66.2% to 91.3% in diverse populations.^34^, ^35^, ^66^ One study interviewed parents of youths with mental health conditions and identified trust, clarity and flexibility of the research process as key factors influencing participation.^67^ Strategies to promote sensitive hair collection procedures and maximise participant engagement include clear and respectful introductions and explanations of the reasoning behind hair collection, a comfortable and safe environment, parental involvement, flexible hair collection strategies/occasions and considerations of developmental and cultural preferences.^34^, ^68^ When discussing these issues with our YPAG, they did not raise major concerns about the EMA and indicated up to four prompts (EMA measurement instances) per day would be manageable, that having a wide window to respond, being told that they can miss some prompts if needed, having an offline data collection option and reminders to complete pending prompts would be helpful. They noted the challenges of responding during school time, both because of the school schedule and because they may not feel comfortable responding in the presence of peers for sensitive questions. On the other hand, they felt that the on‐boarding, online survey and hair sampling components may be easier to do during a dedicated time at school. They also raised some concerns about location tracking, which they indicated might feel intrusive.

More broadly, they indicated that the biggest barrier to participation might be having to commit to 5 years, that participation would be more difficult during exam times, that parents' involvement could be a barrier and that confidentiality assurance would be very important (especially in relation to sensitive components such as hair sample analyses and location tracking). The discussions also indicated that explanations of confidentiality should assume little prior knowledge of the research process, as young people may not assume that they will be responding anonymously or that their data will be confidential. Co‐design of the research materials with young people can help ensure that these materials are clear for prospective participants.

The YPAG also provided some information on factors that they did not consider to be significant barriers. For example, they were happy with the idea of providing hair samples and suggested that they would not have concerns about answering questions on sensitive topics. Strategies related to reducing barriers and burden thus included selecting easy‐to‐use software and hardware with minimal burden, including an easy‐to‐use smartphone app for participants' own phones and nonwearable sleep‐based measuring devices; clear explanations of confidentiality and anonymity (especially for any particularly sensitive components such as biosampling or location tracking); allowing adolescent‐only participation (i.e., parental participation is not mandatory); avoiding data collection at exam times; wide windows allowed to respond to EMA prompts; instructing participants to aim to answer 3–4 EMA prompts per day, but sending 8 prompts to provide them with ample opportunities to respond and making it clear that it is OK to miss some prompts (e.g., in school time); seeking to negotiate a time to collect data within schools (if possible) and renewing participation commitments on an annual basis rather than requesting a 5‐year commitment up‐front.

### 2.5 Other considerations

There remains much to be learned about how to optimise recruitment and retention/adherence in EMA studies, especially measurement burst studies and in adolescence.^9^ To contribute to building knowledge of effective strategies, our study publication plan includes analyses of adherence and attrition rates and momentary and person‐level predictors of adherence and attrition. Though there has been limited research on this to date, evidence suggests that EMA adherence is predictable based on personal and momentary characteristics, as well as earlier EMA engagement patterns.^58^, ^69^, ^70^, ^71^ The predictability of adherence opens up the possibility of adaptive designs or ‘just in time’ momentary interventions that aim to tailor protocols based on for whom and when there is a risk of nonengagement; however, research suggests that the extent to which adherence can be predicted is modest and more needs to be learned about how to gather and leverage data that can predict and intervene on disengagement more effectively. To this end, we will also include questions about the perceived burden of study components and ask participants about their reasons for dropping out to gain further insights into specific intervention points to improve participation in future studies.

While high and nonselective recruitment/retention is ideal, some nonparticipation is inevitable; therefore, to mitigate bias, we will implement statistical approaches to address missing data such as multiple imputation, nonresponse/attrition weighting, Bayesian estimation and full information maximum likelihood (all with relevant auxiliary variables) to provide unbiased parameters under a ‘missing at random’^72^ assumption. NMAR (not missing at random) methods will also be considered as relevant.

### 2.6 Limitations

It is important to acknowledge the limitations of our engagement strategy. First, some nonrandom, nonresponse is unavoidable; therefore, the strategy is expected to reduce but not eliminate all sources of bias related to nonresponse. Second, while we conducted YPAGs to input on the engagement plan and members were sampled to be as diverse as possible, these inevitably gathered the input of only a small number of young people whose views do not necessarily reflect those of all prospective study participants. There is evidence that YPAGs tend to show a degree of self‐selection^73^, ^74^ and their views may therefore reflect those of young people who are more likely to be inclined to be engaged with research. Third, while we based our strategies on a comprehensive review of the literature, there are some areas where evidence is lacking and there is no specific evidence available on effective engagement strategies for longitudinal multimodal EMA burst studies like MHIM. Fourth, due to the longitudinal nature of the study and need for consistency of data collection protocols over time, the developmental tailoring of the engagement strategy will be limited to relatively minor variations such as the language used and design‐based aspects of study materials. Finally, the strategy developed does not include all possible components that could promote engagement as it must consider both resource constraints and be balanced against the potential negative consequences of some strategies. For example, we will not offer comprehensive individual‐level feedback on participants’ responses because this could induce behaviour change and undermine the observational nature of the study. Instead, we will focus on providing feedback in forms (e.g., appealing visuals rather than detailed personalised narratives) and in domains (e.g., on response rates) where the risk of substantial measurement reactivity is reduced.

## 3 CONCLUSIONS

Engagement strategies for adolescent studies should be based on current evidence and consultations with adolescents on effective strategies. We outline the key components (in the domains of co‐production, incentives, relationships and barrier reduction) of the MHIM engagement strategy and present background evidence motivating the inclusion of the components.

## AUTHOR CONTRIBUTIONS

**Aja L. Murray**: Conceptualisation; methodology; supervision; project administration; writing—review and editing; writing—original draft; funding acquisition. **Tong Xie**: Writing—original draft; writing—review and editing. **Luke Power**: Writing—review and editing. **Lucy Condon**: Project administration; writing—review and editing.

## CONFLICT OF INTEREST STATEMENT

The authors declare no conflict of interest.

## Supporting information

Supporting information.

## Data Availability

Data sharing is not applicable to this article as no new data were created or analysed in this study.
