# Erratum to “Recruitment and Retention of Adolescents for an Ecological Momentary Assessment Measurement Burst Mental Health Study: The MHIM Engagement Strategy”

**DOI:** 10.1111/hex.70117

**Published:** 2024-11-28

A. L. Murray, T. Xie, L. Power, and L. Condon, “Recruitment and Retention of Adolescents for an Ecological Momentary Assessment Measurement Burst Mental Health Study: The MHIM Engagement Strategy,” *Health Expectations* 27, no. 3 (2024): e14065.

In the author information, the credentials of Luke Power were incorrectly listed as ‘MSc’ and should be corrected to ‘MA’. Additionally, the credentials of Tong Xie were incorrectly listed as ‘MSc’ and should be corrected to ‘BA’.

We apologize for this error.

