# Supplementary material for: Recruitment and retention of adolescents for an ecological momentary assessment measurement burst mental health study: The MHIM engagement strategy
Source: Health Expect. 2024 May 6;27(3):e14065. doi: 10.1111/hex.14065 (PMC11074385; doi:10.1111/hex.14065)
Supplement: Supplementary file 1 — Supporting information. [file HEX-27-e14065-s001.docx]

**Table S1: Views expressed by young people and how they influenced the study**

| **View (age group)** | **Response** |
| --- | --- |
| They would generally be interested in taking part (both groups) |  |
| They would want taking part to be as easy as possible (11-13) | The protocol will be reviewed with young people to identify ways to make it as easy as possible to take part. |
| Five years seems like a long time commitment (both groups) | The project will ask young people to commit one year at a time. |
| Learning about mental health and stress would motivate them to take part (both groups) and these topics were seen as relevant for teenagers (both groups) | The project will include several ways in which to provide information to young people about mental health and stress, through sessions offered at the school/class level and through embedding mental health facts in the study flow. |
| They would like to learn about their own mental health and stress through individual-level feedback (11-13) | It was explained that this would be difficult because it might undermine the observational nature of the study and because it is not scientifically valid to interpret the meaning of the data at the individual level. |
| Advertising the study through schools might make it seem boring by association with school/school work. On the other hand it would give it legitimacy and they would trust is more if it came via school. If advertised via social media it would need to be made clear that it was a legitimate study (11-13) | On balance advertising the study through schools appeared to have more advantages than disadvantages, therefore, this approach will be adopted. |
| Young people will not assume that the data is confidential and in general a good understanding of how this works in research and these issues should not be assumed. It should be explicitly pointed out that their responses won’t be seen by parents (14-17) | Explanations of concepts like confidentiality will be clearly explained and the explanations of the research, including these aspects will be co-produced with young people. It will be explicitly explained that parents will not see young people’s responses. |
| Young people had concerns about their location being tracked as part of the EMA component of the project (14-17) | The fact that location will not be tracked in real time will be made very clear to young people. Communications around this component of the project to prospective participants will be co-produced with young people to make sure any concerns are addressed. This component of the project may be dropped after further consultations with young people if it is felt that their concerns cannot be adequately allayed and replaced with self-report questions about their location (e.g., self-reported amount of green space). |
| Young people might not be able to respond to all EMA prompts immediately (14-17) | Young people will be given a long window to respond (scaled to the length of the data collection window but potentially up to 2 hours). |
| Having to do exams will make it harder to take part in the study (14-17) | We will schedule the data collections with the goal of avoiding exam and other busy periods. |
| Young people were happy to answer sensitive questions as long as it was confidential (14-17) | We will include questions on sensitive topics (e.g., sex and relationships) as intended in the original project proposal. |
| Young people would be more likely to take part if the invitation came via the parents (11-13) | The study is already planned to engage parents via collecting parental consent (as relevant) and by inviting them to take part in parent surveys. We will additionally aim to engage parents in other ways, such as creating a parent section of the website to maximise their buy-in. |
| Young people may want to know about the background to the research, why it was taking place, and who the researchers/institutions doing it were (11-13) | In the study materials/website we will include this information, including profiles for the team members. |
| Having flexibility in when data collections can be completed would be helpful. On the other hand, a specific time scheduled might make it easier for people to take part (14-17) | We will seek to have some of the data collection take place within schools at specific times; however, multiple timeslots will be available to provide flexibility. Other aspects of the data collection can be completed more flexibly such as the online surveys, will follow-up reminders issued to help young people to remember to complete it. |
| Allowing offline data collection would make completing the data collection easier (14-17) | We have selected an EMA app that will permit offline data collection. |
| Email/text reminders could help young people to remember to complete the data collections (14-17) | Reminders will be issued to those who have not yet completed the data collection. |
| Participating during school time during lessons would mean that young people did not have to give up any of their free time (11-13). On the other hand, they might feel less comfortable responding to sensitive questions at school (14-17) | We will seek to complete some of the data collection in school to minimise the burden for participants, ensuring that the setting allows people to feel comfortable responding; however, we will make it clear that other aspects (e.g., responding to EMA prompts during school time as opposed to out of school hours) are not mandatory and the incentive given will not be affected by the response rates of EMA prompts during school hours. |
| There was debate about whether school holidays were a time where people might be more or less likely to take part (11-13). As young people may be given homework to complete over the holidays framing participation like this as homework might make it more likely that they would complete it (11-13) | We will not specifically avoid data collection during the holidays but will have a flexible enough data collection timeline and reminder schedule to accommodate the fact people may be away and/or less engaged during these periods. |
| For the EMA, the range of manageable prompts per day was 2-4 (both groups) | Young people will be told that their aim is to complete 3 prompts per day. |
| There were differences of opinion about whether young people should be provided with an incentive as it might mean that people provide low quality data just to receive a reward (11-13) but an incentive would be expected/fair, would make people feel valued, and should be scaled to the level of participation (14-17) | On balance, the advantages of providing incentives seemed to outweigh the disadvantages discussed, therefore, shopping vouchers will be offered to participants. The amount of incentive will be increased if participants take part in more components of the research and respond to more EMA prompts outside of school hours. |
| Certificates might not motivate people to take part in the first place but might be good for retention (14-17) | Certificates will be provided for completing key milestones in the data collection. |
| Keeping the sleep devices could be motivating (14-17) | Those who complete the entire data collection cycle will be allowed to keep the sleep devices. |
| A session on how to write up study participation in a CV would be motivating (14-17) | Young people will be provided with advice on how they could reflect their research participation in CVs, personal statements, and interviews. |
| School-level feedback might help motivate young people to take part but not as much as individual-level feedback (14-17) | Schools in which a minimum number of pupils take part will be provided with school-specific feedback. |
| Young people would be interested to know who the researchers are initially e.g., via presenting at assembly but felt it would be less important to have contact after that (both groups). Being able to have a back & forth conversation with the researchers might help young people feel valued (14-17) | We will seek to visit schools initially to present the project and encourage participation and answer participant queries. If not possible, online Q&A sessions will be set up. The researchers will be approachable by email. |
| If the researchers were friendly and if they liked the researchers, this might influence how likely they were to take part (14-17) | Research staff will be trained in how to engage with young people and make them feel valued. |
| Participants might feel more valued if it was shown how the information they are providing is impacting on our understanding of mental health (14-17) | This will be clearly articulated in the study materials. |
| Young people might be more likely to take part in a study that they knew other young people had been consulted on (14-17). They felt that consultation on the measures and findings were the two most important aspects to consult young people on (14-17) | The fact that the study is being conducted with young person advisors will be made clear in the recruitment/participation materials. |
